# Multi-Source Data-Driven Estimation Model for Passenger Flow Management in Urban Rail Transit

**DOI:** 10.3390/s26165093

**Published:** 2026-08-11

**Authors:** Kaiwen Hou, Zhengping Tao, Yongtao Liu, Jiankun Yuan, Jianfan Wu, Kai Yu

**Affiliations:** 1 Department of Traffic and Transportation, Beijing Jiaotong University, Beijing 100044, China; 2 CETC Big Data Co., Ltd., Guiyang 550081, China; 3 School of Automobile, Chang’an University, Xi’an 710064, China

**Keywords:** multi-source data, passenger flow estimation, data fusion, urban rail transit

## Abstract

The accurate estimation of real-time passenger flow in urban rail transit (URT) networks plays a crucial role in optimizing the operation and management of urban rail transit systems, with profound implications for daily operation scheduling, passenger flow control, and safety management. Most existing studies have relied solely on a single data source such as Automatic Fare Collection (AFC) data for real-time passenger flow estimation. However, the inherent data uploading delay in the urban rail transit AFC system often leads to the delayed acquisition of passenger flow information. This severely limits the timeliness and accuracy of passenger flow estimation, thereby affecting the efficiency of URT operation and management. To address this critical challenge, this study proposes a multi-source data-driven estimation model to achieve the fusion of multi-source heterogeneous data covering the uploaded AFC data, historical passenger flow data and mobile phone signaling data collected from mainstream sensors such as through-beam photoelectric sensors and RFID/NFC sensors. In the proposed model, various types of information are taken into account by extracting features of the different data sources. The advantages of the proposed model are validated by utilizing multi-source data from Chengdu, China. The experimental results demonstrate that the proposed model achieves higher accuracy compared to existing benchmark models. Compared with the second-best-performing model, it reduces the MAE by 8.1%, RMSE by 10.7%, and MAPE by 17.2% at the 15 min time granularity, which shows that the proposed model has effective performance in terms of accuracy and stability.

## 1. Introduction

Driven by accelerating urbanization and burgeoning demand for high-efficiency, eco-friendly, and convenient transportation modes, urban rail transit (URT) plays an increasingly pivotal role in public transportation systems. As a green and high-capacity means of transportation, URT not only effectively alleviates urban traffic congestion and reduces environmental pollution caused by motor vehicles, but also promotes the reasonable distribution of urban functional areas and the integrated development of urban and suburban areas, thus exerting a profound influence on the optimization of the operation efficiency of urban public transportation networks and improving the quality of urban life. Specifically, the estimation of real-time passenger flow in urban rail transit networks is indispensable in the operation and management of urban rail transit systems. As a crucial component of real-time passenger flow estimation, short-term passenger flow prediction (STPFP) holds fundamental significance for evaluating the operational state of URT systems and improving their management and service levels [1,2,3,4,5,6].

Traditional approaches for passenger flow prediction are mainly mathematical and statistical models such as the ARIMA models [7,8], Kalman filtering models [9,10], and linear regression models [11]. For instance, Guang et al. [12] adopted the seasonal ARIMA (SARIMA) to forecast the short-term passenger flow of urban railway stations. Kumar et al. [13] employed a forecasting method utilizing a seasonal ARIMA model for the short-term prediction of traffic flow. Nevertheless, statistical-based models for passenger flow prediction are unable to effectively capture the complex nonlinear relationships within time series, which results in limited adaptability and performance.

With the rapid development and application of machine learning techniques, models based on machine learning have been widely applied in passenger flow prediction, such as random forest [14,15], back propagation neural networks [16] and support vector machines (SVMs) [17,18]. For instance, Sun et al. [19] introduced a wavelet–SVM method to predict the short-term passenger flow of the Beijing metro system. Wang et al. [20] proposed a partially online SVM model to capture the periodicity and nonlinearity characteristics of short-term passenger flow.

However, these models possess inadequate capabilities in modeling time series and are consequently not accurate enough for robust time series prediction.

In the past few years, different kinds of deep learning models such as LSTM, CNN, RNN, and Transformer [21,22,23,24] have been successfully applied to traffic flow prediction owing to their powerful and effective learning capability in capturing temporal information. Wu et al. [25] proposed a spatial–temporal hypergraph attention recurrent network (STHGARN) to enhance the accuracy of STPFP. Zhang et al. [26] proposed a deep learning architecture called Conv-GCN to tackle the STPFP problem. Li et al. [27] employed a learning-based prediction network called an improved CNN-LSTM framework with similarity grouping to predict vessel traffic flow (VTF) and then handle the issue of traffic state estimation (TSE) under the scenario of data sparsity. Du et al. [28] proposed a graph ensemble deep random vector functional link network (named GEdRVFL) and used it for node-specific traffic flow prediction. Liang et al. [29] adopted a spatiotemporal multi-graph convolutional network (STMGCN)-based vessel traffic flow prediction method by exploiting various types of inherent correlations in the generated maritime graph. Huang et al. [30] proposed a hybrid short-term metro passenger flow prediction model named decomposition ensemble attention sequence to sequence (DEASeq2Seq) to conduct STPFP. Yang et al. [31] established a multi-task learning-based model called Res-Transformer for STPFP with multiple traffic patterns. Zhang et al. [32] built a Transformer-based architecture called COV-STFormer, which is within an encoder–decoder framework, to achieve accurate passenger flow prediction during the COVID-19 pandemic. Xie et al. [33] constructed a patched Transformer-based sequence-to-sequence model named MultiSize Patched Spatial–Temporal Transformer Network (MSP-STTN), which is utilized to incorporate rich and unified context modeling through a self-attention mechanism and global memory learning via a cross-attention mechanism for short- and long-term grid-based crowd flow prediction. Zhang et al. [34] introduced a TSE model, which combined the computational graph with physics-informed deep learning (PIDL) methods. Bapaume et al. [35] constructed a computer vision framework based on deep learning techniques to enable the real-time prediction of passenger load and train schedules in urban transportation networks specifically for metro lines. Zeng et al. [36] established a spatial–temporal dynamic Transformer-based model for passenger flow prediction in urban rail transit. As summarized above, deep learning models have been extensively adopted for STPFP tasks. Nonetheless, existing research still focuses less on utilizing multi-source data and may not be applicable to partial scenarios demanding multi-source data.

For multi-source data fusion, current methods generally utilize normal continuous historical data for predictions [37,38]. However, such approaches often integrate weakly correlated information with different travel patterns. More specifically, there are essential disparities existing in passengers’ travel behaviors between weekdays and weekends. Failure to distinguish these differences may bring noise into the prediction process, which causes potential inaccuracy. To comprehensively capture spatial–temporal features in STPFP tasks, a number of recent studies have proposed hybrid architectures and leveraged multi-source data, which can improve the accuracy of prediction and robustness of the model. Generally, distinct neural networks are adopted for feature extraction from different data sources, where the feature extraction is a crucial part in multi-source data fusion. For example, Geng et al. [39] used the multi-GCN to extract spatial features and a contextual gated RNN to analyze time series data. Zhao et al. [40] integrated the GRU and GCN where the GRU is applied to extract temporal dynamic changes and the GCN is used to learn topological dependencies. Zhang et al. [41] introduced ResLSTM, which is based on ResNet, GCN, and LSTM. The introduced ResLSTM also considers weather and air conditions, which will exert an influence on passengers’ travel plans, and uses residual connections to prevent gradient vanishing while reserving more low-level information. Li et al. [42] introduced an attention mechanism and employed a multi-head GAT and LSTM to conduct traffic forecasting. Cheng et al. [43] proposed a hybrid subway passenger flow forecasting approach, which adopts a multi-source data fusion technique in consideration of the deficiency of existing research that mainly focuses on feature selection while ignoring effective feature fusion. Chen et al. [44] proposed a multi-stage fusion passenger forecasting (MSFPF) model to achieve short-term multi-step passenger forecasting leveraging multi-source data in a URT network. Ma et al. [45] proposed a Large Language Model (LLM)-based metro passenger flow prediction method, incorporating hyperparameter tuning, pre-training, and multi-source spatiotemporal data fusion to increase the accuracy of prediction. Moreover, Tao et al. [46] proposed the online estimation model and the error layered optimization algorithm to implement the estimation of the present passenger flow state based on multi-source data. Bai et al. [47] combined meteorological data for passenger demand forecasting and processed it properly. All of these studies considered multi-source data where spatial–temporal features together with some external influencing features could be obtained to promote prediction accuracy. Consequently, exploring the effective and reasonable approaches to fuse multi-source data has become an essential topic. Considering the heterogeneity of multi-source data and the complicatedness of multi-source heterogeneous data fusion, the automatic differentiation (AD) method [46,48] is introduced as the framework and is modified to achieve the fusion of multi-source data.

## 2. Research Background

### 2.1. Notations and Problem Statement

In this section, the corresponding notations are listed in Table 1.

### 2.2. Problem Statement

AFC data (namely card-swiping data) is mainly acquired by the through-beam photoelectric sensors installed in the automated fare gates of urban rail transit. These sensors are the fundamental hardware carriers of passenger flow perception and the study is developed based on the actual sensing data generated by these sensing devices. There exists an inherent time delay during AFC data transmission, caused by the data uploading interval from automatic ticket gates to the Automatic Fare Collection Clearing Center (ACC), which undertakes AFC data processing. This transmission interval normally ranges from 15 min to one day. As a result, urban rail transit operation and management departments are unable to gain access to network-wide passenger flow data in a timely manner. Furthermore, two categories of AFC data are formed in the uploading process: one merely covers passengers’ inbound information while the other contains full information. AFC data with only passengers’ inbound information can be collected and transmitted to the ACC system immediately when passengers swipe cards to enter stations. In contrast, AFC data with full information is only accessible after passengers complete their trips across the urban rail transit network. To ensure estimation accuracy, AFC data with only passengers’ inbound information is utilized to conduct OD distribution estimation and path matching, so as to derive estimated passenger flow indicators. Once complete trip-based AFC data becomes accessible, it is used to calibrate the preliminarily estimated passenger flow indicators. Additionally, for time intervals where AFC data cannot be acquired timely, the inbound quantity of passengers is firstly predicted, followed by OD distribution estimation and path matching operations. Accordingly, the passenger flow estimation for the corresponding time interval can be largely accomplished.

However, the estimation results derived solely from a single data source are still inaccurate. One of the major challenges in passenger flow prediction lies in the heterogeneity, noise, missing values, and spatiotemporal inconsistency of data acquired from different kinds of sensors. It is critical to leverage the complementary advantages of multi-source data collected from different kinds of sensors and handle a single data source’s redundancy and distortion. Thus, it is necessary to estimate the present passenger flow across different spatial and temporal dimensions such as the network and obtain the estimated passenger flow indicators based on multi-source data acquired from different kinds of mainstream sensors to enable the URT management department to learn about the dynamic passenger flow distribution in the urban rail transit network promptly and precisely. In this way, the estimation outputs closely match the real-time dynamic passenger distribution across the network.

### 2.3. Data Source Introduction

As mentioned above, Automatic Fare Collection (AFC) data, also known as card-swiping data or smart card transaction data, represents passenger tap-in and tap-out swiping logs at metro stations. The mobile phone signaling data refers to anonymized cellular network event records passively generated by user–terminal interactions with communication base stations, featuring full urban spatial coverage and minute-level temporal resolution. As a critical complementary data source to AFC data, it can reconstruct complete passenger travel chains covering pre-metro access, in-station riding and post-metro egress, and support the extraction of city-wide dynamic OD matrices. Nevertheless, mobile phone signaling data carries inherent limitations: it only provides coarse base station-level positioning without precise user coordinates, and suffers from ping-pong handover noise and position drift anomalies. Furthermore, the count of SIM subscriptions cannot be directly converted to actual passenger numbers due to multi-card ownership and demographic penetration bias, and raw signaling lacks explicit travel mode labels to distinguish metro riders from other passengers. Therefore, spatial map matching, temporal synchronization, bias correction, unified 15 min passenger flow aggregation, Min–Max normalization, and multi-source fusion with AFC data are indispensable preprocessing steps before using mobile phone signaling data for URT passenger flow state estimation (passenger flow state herein mainly refers to the real-time spatiotemporal distribution of passengers within the URT network). The historical passenger flow data is constructed by long-term archived Automatic Fare Collection transaction records aggregated at a 15 min time granularity. This type of data possesses prominent multi-level periodic temporal features including daily commuting cycles and weekly weekday–weekend differentiation, and supports hierarchical spatial aggregation across the network, line, section, station and OD pair layers matching the topological structure of the Chengdu URT network. Fully reconciled and cleaned historical passenger flow data acts as reliable ground-truth priors, supplying stationary baseline inbound/outbound passenger quantity, OD selection ratios and route selection ratios as core prior variables for the proposed multi-source fusion model. However, historical passenger flow data suffers from inherent static lagging defects: it fails to capture real-time abrupt passenger fluctuations and off-street travel trajectories, and loses validity when temporary network adjustments take place. Complementary fusion with uploading AFC data and mobile phone signaling data can effectively offset these drawbacks and enhance the accuracy and timeliness of network-level passenger flow state estimation.

## 3. Methods

### 3.1. Passenger Flow Estimation Model

The model for the estimation of present passenger flow is established in this part. Different types of data sources, namely AFC data, historical passenger flow data and mobile phone signaling data, are fused to obtain locally optimal estimated values of different variables at different levels based on the AD framework. This part constructs corresponding mean square error loss functions (respectively $F_{1}(\alpha )$, $F_{2}(\gamma )$, $F_{3}(\beta )$, $F_{4}(\upsilon )$ and $F_{5}(\eta )$) based on five estimation outputs: inbound quantity, selection ratios of different OD pairs, selection ratios of different routes within each OD pair, sectional passenger flow, and outbound quantity. By considering the integrity of the estimation process of passenger flow and integrating the error functions as the objective function F(α,γ,β,ν,η) of passenger flow estimation, the correlations between various data sources and estimated variables and parameters are presented as follows. Here, *N1*, *N2*, *N3*, and *N4* denote the sets of stations, OD pairs, travel routes, and sections respectively.

(1) $$F_{1}(\alpha )=\frac{1}{N_{1}}({\sum_{o=1}^{N_{1}}\left(X_{o}^{t}-\bar{X_{o}^{t}}\right)}^{2})$$

(2) $$F_{2}(\gamma )=\frac{1}{N_{2}}({\sum_{ow=1}^{N_{2}}\left(P_{ow}^{t}-\bar{P_{ow}^{t}}\right)}^{2})$$

(3) $$F_{3}(\beta )=\frac{1}{N_{3}}({\sum_{wr=1}^{N_{3}}\left(P_{wr}^{t}-\bar{P_{wr}^{t}}\right)}^{2})$$

(4) $$F_{4}(\upsilon )=\frac{1}{N_{4}}({\sum_{a=1}^{N_{4}}\left(X_{a}^{t}-\bar{X_{a}^{t}}\right)}^{2})$$

(5) $$F_{5}(\eta )=\frac{1}{N_{1}}({\sum_{o=1}^{N_{1}}\left(Z_{o}^{t}-\bar{Z_{o}^{t}}\right)}^{2})$$

where $F_{1}(\alpha )$ denotes the deviation between the station-level inbound quantity based on historical passenger flow data and the estimated inbound quantity of the station generated by the model. $F_{2}\left(\gamma \right)$ denotes the deviation between the proportion of the origin station O to all the destination stations D obtained by historical passenger flow data and the estimated proportion of the origin station O to all the destination stations D obtained by the model. $F_{3}(\beta )$ denotes the deviation between the selection ratio of different routes from the origin station O to the destination D based on mobile phone signaling data and the estimated selection ratio of different routes from the origin station O to the destination D obtained by the model. $F_{4}\left(\upsilon \right)$ denotes the deviation between the passenger flow of the section obtained by historical passenger flow data and the estimated passenger flow of the section obtained by the model. $F_{5}(\eta )$ denotes the deviation between the station-level outbound quantity based on historical passenger flow data and the estimated outbound quantity obtained by the model.

Hence, the objective function could be formulated to achieve minimizing the above mean square error loss functions:

(6) $$\min F\left(\alpha ,\gamma ,\beta ,\nu ,\eta \right)=F_{1}\left(\alpha \right)+F_{2}\left(\gamma \right)+F_{3}\left(\beta \right)+F_{4}\left(\nu \right)+F_{5}\left(\eta \right)$$

**Figure 1:**
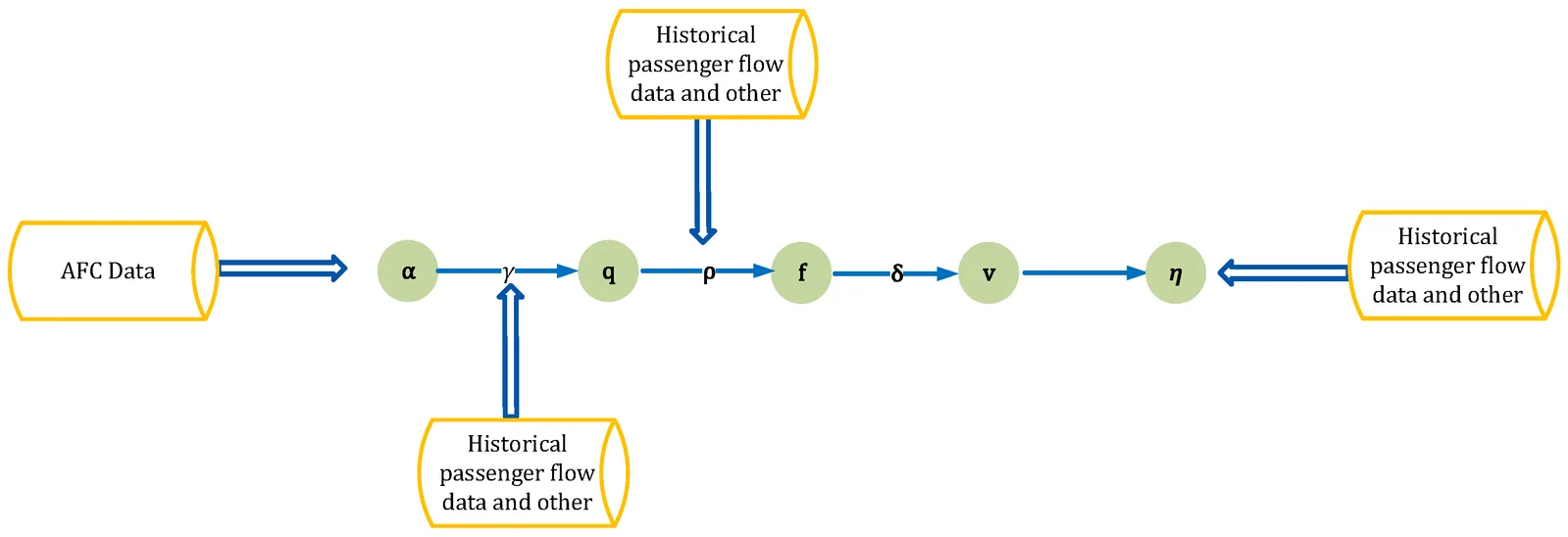
Brief diagram of data fusion and estimation process.

**Figure 2:**
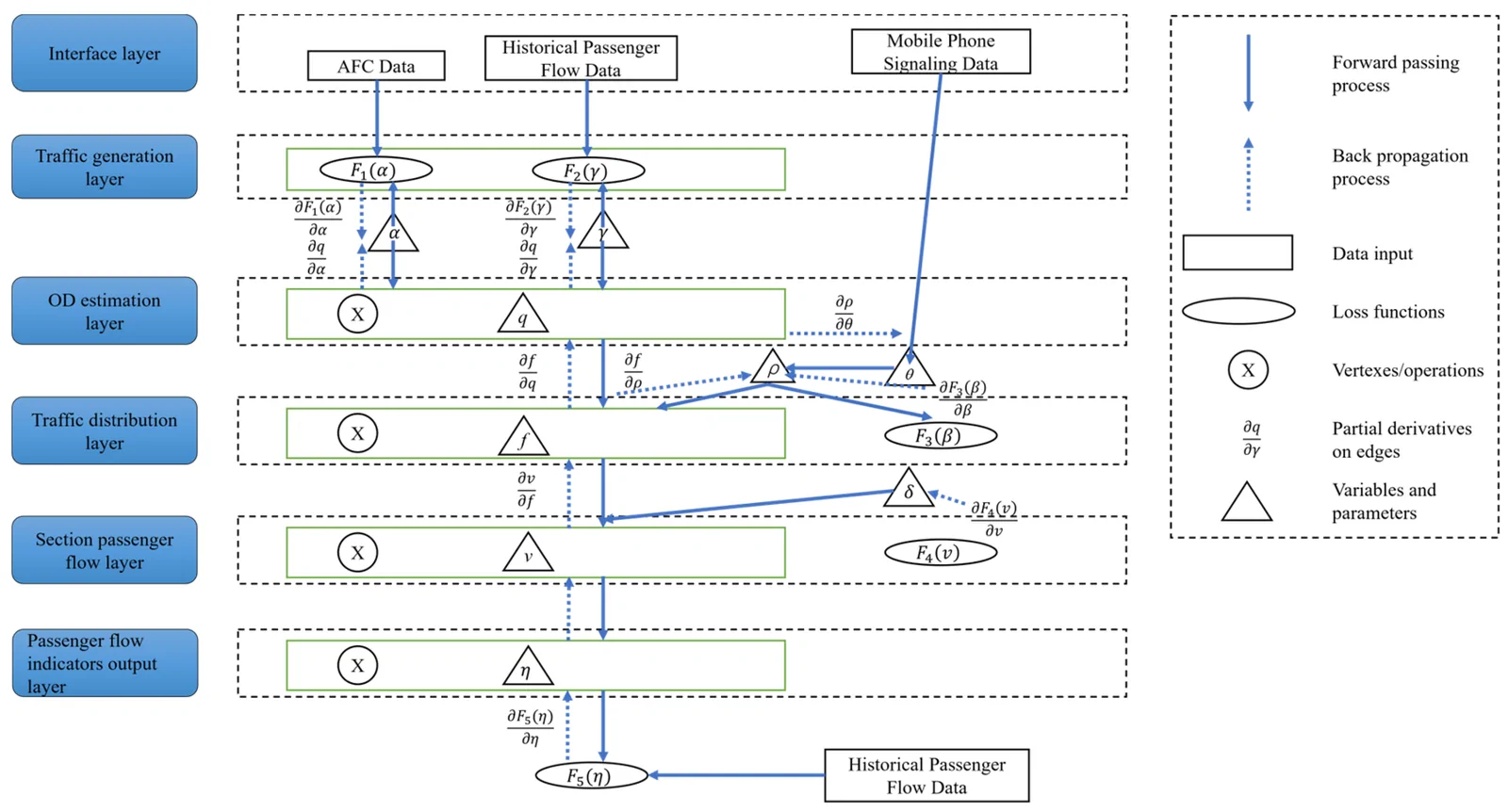
Illustration of detailed estimation process.

Equation (6) indicates that the optimization objective minimizes the aggregate loss of all five estimated loss functions. Figure 1 briefly illustrates the representation of multiple data sources being fused to the estimation process (“other” in Figure 2 only refers to the mobile phone signaling data). The variables tabulated in Table 1 can be rearranged into vectorized forms, with the vector definitions specified as follows: inbound quantity $\alpha =\left(X_{o}^{t}|o\in V\right)$; OD quantity $q=\left(X_{w}^{t}|w\in W\right)$; path quantity $f=\left(X_{r}^{t}|r\in R\right)$; section quantity $v=f\left(X_{a}^{t}|a\in E\right)$ and outbound quantity $\eta =\left(Z_{o}^{t}|o\in V\right)$. Furthermore, the OD selection ratio $\gamma =\left(P_{ow}^{t}|o\in V,w\in W\right)$ and path selection ratio $\rho =\left(P_{wr}^{t}|w\in W,r\in R\right)$ are derived through normalization. Among them, v is the dependent variable of f; f is the function of q and ρ; q is the function of α and γ; γ is the function of $\pi_{ow}^{t}$; ρ is the function of t; and η is the function of α, β, and γ.

The following passenger flow conservation equation must be observed in modeling:

(7) α×γ=q

(8) q×ρ=f

(9) f×δ=v

Moreover, the constraints imposed on the objective function F(α,γ,β,ν,η) are presented below:

(1) Recurrence relation of passenger flow

(10) $$X_{o}^{t}P_{ow}^{t}=X_{w}^{t},\forall o\in V,\forall w\in W$$

(11) $$X_{w}^{t}P_{wr}^{t}=X_{r}^{t},\forall w\in W,\forall r\in R$$

(12) $$\sum_{r\in R}X_{r}^{t}\delta_{ra}=X_{a}^{t},\forall r\in R,\forall a\in E$$

where Equation (10) denotes that the product of the estimated inbound quantity $X_{o}^{t}$ at station O and the OD selection ratio $P_{ow}^{t}$ is equal to the estimated OD estimation quantity $X_{w}^{t}$; (11) denotes the product of a certain OD estimation quantity $X_{w}^{t}$ and the selection ratio $P_{wr}^{t}$ of different routes in the OD is equal to the estimated passenger flow of a certain route $X_{r}^{t}$; and (12) denotes that the product of the estimated passenger flow of a certain route $X_{r}^{t}$ and the coefficient $\delta_{ra}$ is equal to the estimated passenger flow on the section $X_{a}^{t}$.

(2) Selection probability of passenger travel

After normalizing the selection ratio $\pi_{ow}^{t}$ of the starting station to a certain destination station derived from model-based calculations, the standardized selection ratio $P_{ow}^{t}$ of OD to $\pi_{ow}^{t}$ is obtained:

(13) $$P_{ow}^{t}=\frac{\pi_{ow}^{t}}{\sum_{w\in W}\pi_{ow}^{t}}$$

Passengers choose travel paths with inherent randomness. Since there are multiple travel paths to choose from in a certain OD pair, the problem of passenger travel path selection can be modeled as the path probability selection problem. Therefore, the probability that the *r*th effective path between the OD pair *w* is selected could be obtained:

(14) $$P_{wr}^{t}=\frac{\exp\left(-t_{o}^{in}-t_{a}-\delta_{rtran}\theta t_{o}^{tran}-t_{o}^{out}\right)}{\sum_{r\in R_{w},a\in E,o,d\in V}\exp(-t_{o}^{in}-t_{a}-\delta_{rtran}\theta t_{o}^{tran}-t_{o}^{out})}$$

The probability of selecting each valid travel path follows the property that the sum of all path selection probabilities equals 1. This constraint guarantees normalized probability outputs. Therefore, path selection probabilities satisfy the following boundary conditions:

(15) $$0\leq P_{wr}^{t}\leq 1$$

(16) $$\sum_{r\in R_{w}}P_{wr}^{t}=1$$

Substituting the constraints (10)–(14) into the objective function $F\left(\alpha ,\gamma ,\beta ,\nu ,\eta \right)$, the new objective function is formulated as follows:

(17) $$\min F\left(\alpha ,\gamma ,\beta ,\nu ,\eta \right)=F\left(\alpha \right)+F\left(\gamma \left(\pi_{ow}^{t}\right)\right)+F\left(\rho \left(t\right)\right)+F\left(v\left(f\left(q\left(a,\gamma \left(\pi_{ow}^{t}\right)\right),\rho \left(t\right)\right)\right)\right)+F_{5}\left(\eta \right)$$

### 3.2. Improved Error Layered Optimization Algorithm

This section presents the improved error layered optimization algorithm (IELOA) comprising the forward passing process and backward propagation process to further implement the estimation process of the URT passenger flow state. Figure 2 illustrates the detailed estimation process, which comprises the forward passing process and backward propagation process.

In the forward passing process, the traffic generation layer utilizes the uploaded AFC data and the estimated parameters of passenger flow to estimate the inbound quantity $X_{o}^{t}$ of each station at different temporal granularities, and generates the error function $F_{1}\left(\alpha \right)$ according to the inbound passenger flow data obtained by historical passenger flow data. The OD estimation layer uses the estimated inbound quantity at each station to multiply the estimated selection ratio $P_{ow}^{t}$ of O to each D, which can obtain the OD quantity $X_{w}^{t}$ of O to each D, and can generate an error function $F_{2}\left(\gamma \right)$ according to the OD travel selection ratio attained by historical passenger flow data. The traffic distribution layer obtains the passenger flow quantity $X_{r}^{t}$ of each route based on the existence of multiple travel routes between the same OD and the traffic distribution selection ratio $P_{wr}^{t}$ obtained by historical passenger flow data, and can generate an error function $F_{3}\left(\beta \right)$ according to the OD travel selection ratio obtained by historical passenger flow data. The section passenger flow layer combines the product of a certain route flow $X_{r}^{t}$ and the binary section passing coefficient $\delta_{ra}$ to obtain the estimated section flow $X_{a}^{t}$, which generates an error function $F_{4}\left(v\right)$ according to the passenger flow quantity of each section obtained by the mobile phone signaling data. Ultimately, the passenger flow indicators output layer generates the error function $F_{5}(\eta )$ based on the outbound passenger flow data obtained by historical passenger flow data. These five sequential calculation steps form the complete forward propagation process for real-time URT passenger flow state estimation.

In the backward propagation process, the FR conjugate gradient method (FRCG) is adopted due to its favorable inherent properties: low memory overhead, global convergence, stable iteration performance, and no need for any external parameters. The partial derivatives of the objective function with respect to all variables are calculated and then the FRCG is employed to solve the search direction and step size for the *N*th back propagation iteration, followed by updating the estimated value of the variables. After variable updating is completed, the algorithm returns to the forward propagation stage and iterates repeatedly until the estimation errors satisfy the predefined convergence criteria. Real-time computation latency is a critical constraint for practical URT operation systems. Accordingly, this study conducts multiple trial estimations to calibrate a reasonable maximum iteration limit. Moreover, the preset threshold is set to 3% based on dataset-based trial calibration. If the objective function loss error exceeds the preset threshold of 3%, the model automatically falls back to historical average passenger flow data as a substitute. In this section, the complete iterative workflow of the improved error layered optimization algorithm is elaborated stepwise as follows:

**Step 1:** Parameter and variable initialization. Input multi-source datasets, and initialize all variables and parameters (for example, $X_{o}^{t}$, $X_{w}^{t}$, $X_{a}^{t}$, etc.).

**Step 2:** Execute the *N*th forward passing process to complete the forward iterative process of all station sets in the URT network. If the error of the objective function exceeds the preset threshold, proceed to **Step 3** to perform *N*th back propagation iteration for all sets. Otherwise, terminate the iterative update process directly.

**Step 3:** Conduct the *N*th back propagation iteration for all sets (stations, OD pairs, paths and sections) to update the values of the variables.

***Step 3.1:*** Determine the search direction of the *N*th back propagation iteration. For all sets of stations, OD pairs, paths, and sections, the partial derivatives of the objective function to all variables at different levels are calculated to construct a negative gradient matrix of variables corresponding to the objective function.

If the iteration index *n* = 1, the search direction *d* is equal to the direction of the negative gradient matrix of the objective function:

(18) $$d_{1}=-\nabla \min F_{1}\left(\alpha ,\gamma ,\beta ,\nu ,\eta \right)$$

Otherwise, the search direction *d* is equal to the combination of the negative gradient matrix direction and the search direction of the previous iteration (where *n* > 1):

(19) $$d_{n}=-\nabla \min F_{n}\left(\alpha ,\gamma ,\beta ,\nu ,\eta \right)+\frac{\left||\nabla \min F_{n}\left(\alpha ,\gamma ,\beta ,\nu ,\eta \right)|\right|}{\left||\nabla \min F_{n-1}\left(\alpha ,\gamma ,\beta ,\nu ,\eta \right)|\right|}d_{n-1}$$

***Step 3.2:*** Determine the step size of the *N*th back propagation iteration. Introduce the undetermined step size variable *λ* into the calculation result $x_{n}$ of the *N*th forward propagation process, then calculate the partial derivatives of the objective function with regard to *λ* to find the value of *λ*.

**Step 4:** Update the values of the variables using the calculated step size *λ*.

(20) $$x_{n}^{\prime }=x_{n}+\lambda d_{n}$$

**Step 5:** Stop conditions.

The two conditions for terminating the algorithm are as follows: (1) the total iteration count reaches the preset maximum iteration limit; or (2) the error of the objective function satisfies the preset convergence threshold. If either condition is met, jump to **Step 6**.

**Step 6:** Output all results.

**Figure 3:**
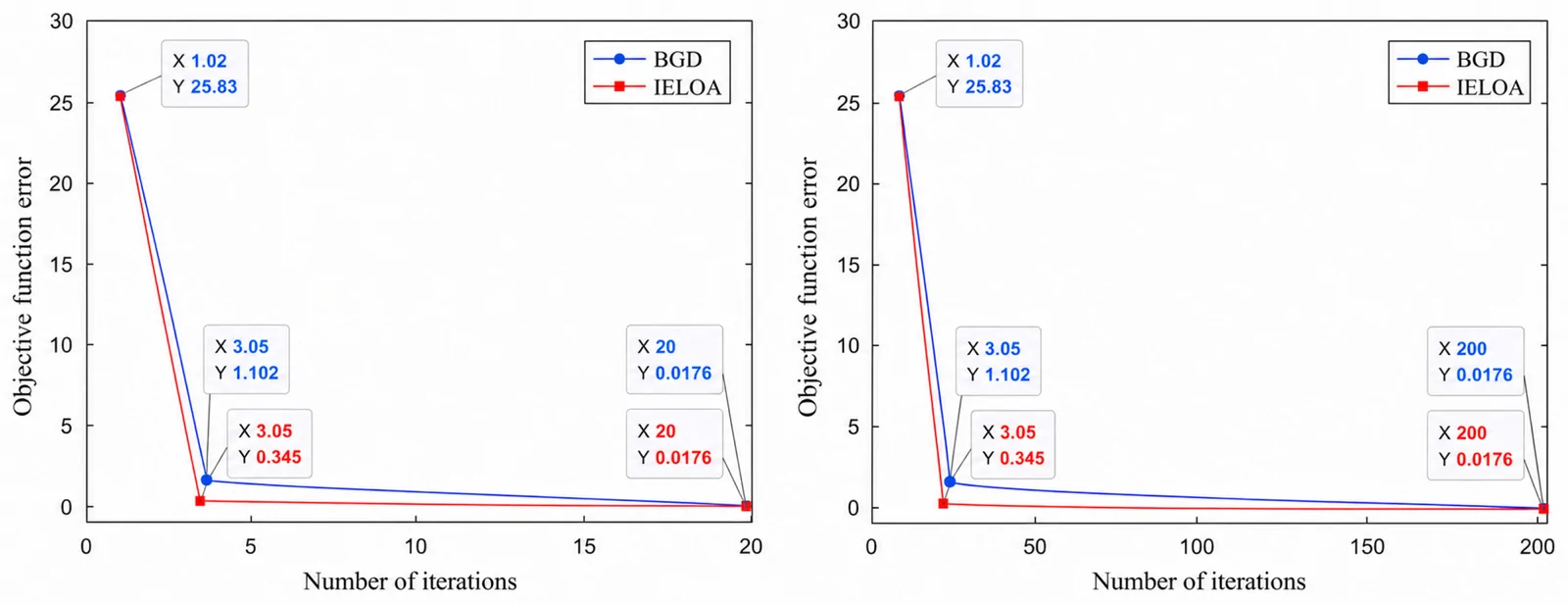
The analysis of the optimization speed of different algorithms.

Practical URT networks typically feature extensive spatial scales, which necessitates systematic verification of the convergence efficiency and numerical accuracy of distinct optimization algorithms. To facilitate this comparative evaluation, we construct a miniature test network consisting of three operational lines (Lines 1, 2, 3) and five independent stations (A, B, C, D, E). After fully implementing the forward propagation process, two algorithms are adopted as comparative benchmarks: the classic batch gradient descent (BGD) and the improved error layered optimization algorithm (IELOA) proposed in this study. All comparative numerical experiments for evaluating the error optimization performance of the two algorithms were implemented using MATLAB R2025b and the computer runs a 64-bit Windows 11 operating system, equipped with an Intel(R) Core (™) i7-1365U processor (1.80 GHz base frequency, up to 4.70 GHz maximum turbo frequency) and 16.0 GB of RAM (Lenovo Group Ltd., Hefei, China). In this comparative experiment, the threshold of the convergence error for iteration termination is predefined as 0.001. As shown in Figure 3, the IELOA exhibits significantly higher error convergence efficiency compared with the BGD method. Therefore, IELOA is selected as the optimization solver to mitigate the cumulative estimation errors produced during the forward propagation process.

## 4. Numerical Experiments

To verify the performance of the developed estimation model using real-world datasets for the estimation of the URT passenger flow state, this part considers using the representative Chengdu metro network as the case study and engineering object. Urban rail transit (URT) is a general transport category covering metro, light rail, tram, automated people mover and other rail transit modes. In the local context of Chengdu, Chengdu Metro refers to the integrated URT network serving Chengdu city and its surrounding areas in China, which is the largest and busiest urban rail transit network in western China. As of 2026, it has 16 operational lines with a total length of 758 km, including tram lines and the Ziyang Line, covering 444 stations (65 transfer stations).

Subsequently, the multi-source data consisting of AFC data, mobile phone signaling data and historical passenger flow data is used and fused through the estimation model for the estimation of the passenger flow state. In this part, the inbound time and outbound time of passengers and other kinds of data are used to help complete the passenger flow distribution process. For the sake of identifying accuracy gaps in the passenger flow estimation between single-source and multi-source data, this study conducts comparative analyses on these two aspects hereinafter.

### 4.1. Passenger Flow Estimation Based on the Single Data Source

This subsection investigates the adoption of single AFC data to deduce dynamic variations in the passenger flow state at stations, sections, lines and the whole network. Since this study mainly centers on passenger flow estimation based on multi-source data, only the network-scale passenger flow estimation indicators are analyzed in this part.

First, this subsection presents the passenger flow estimation results and their corresponding actual data at 9:00 am during morning peak hours from 10 October to 25 October 2018, which were obtained based on a single AFC data source. Table 2 is a comparative analysis of the estimation results and actual values of the passenger flow state of the network under a single data source.

**Figure 4:**
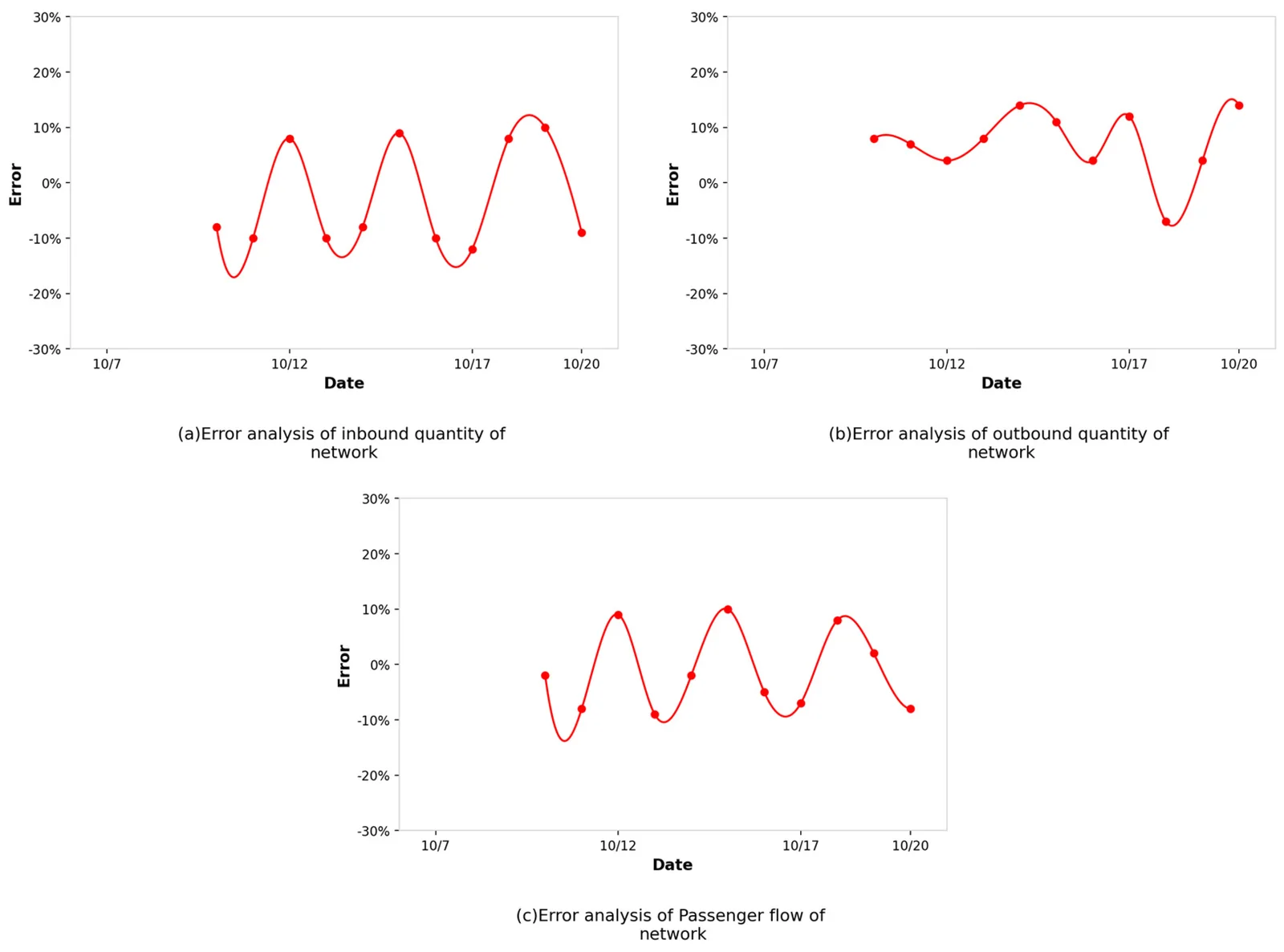
Error analysis of various indicators with single data source.

Figure 4 illustrates the error analysis of various indicators derived exclusively from the single AFC data source. By comparing the errors of various types of indicators from 10 October 2018 to 25 October 2018, it is observed that the errors of the indicators including the network-level inbound quantity, outbound quantity, transfer quantity and passenger flow quantity range from 7% to 10%. The main causes for the relatively large errors in passenger flow estimation with a single data source are summarized as follows. Firstly, data latency exists in AFC data uploading, which makes it impossible to fully obtain complete basic data for analysis and estimation. Secondly, no additional reference samples are available in the indicator estimation process, so the errors introduced by the estimated variables and parameters cannot be promptly and efficiently mitigated.

### 4.2. Passenger Flow Estimation Based on Multiple Data Sources

This section mainly focuses on utilizing AFC data, mobile phone signaling data, and historical passenger flow data to deduce the dynamic variations in the passenger flow state at stations, sections, lines and network. It plays an important role in facilitating the daily management of Chengdu Metro, particularly when commuter passenger flow surges greatly in peak hours.

In view of achieving the passenger flow estimation of the URT network, a topological network model consisting of 156 stations and 24,336 OD pairs is established. To acquire complete network AFC data, AFC sample datasets are retrieved from the official data system of Chengdu Metro during the morning peak hours on 15 January 2019, as illustrated in Table 3. The acquisition of mobile phone signaling data relies on sensing equipment installed across all stations of Chengdu Metro. With the support of these facilities, passengers’ real-time movement trajectories can be tracked, and the collated statistical information is finally uploaded to the system. The passenger flow estimation is implemented within the morning peak hours of 7 am to 9 am and the time interval is set to 15 min.

This part utilizes the proposed method and adopts the dataset ranging from 7:00 to 9:00 on 15 January 2019 to implement passenger flow estimation. The whole calculation took approximately five minutes, during which OD pairs are allocated to different travel paths, and the estimated passenger flow state is renewed every 15 min to derive passenger flow indicators covering different temporal and spatial dimensions.

The network-level estimation results of the passenger flow state, namely the network-level inbound quantity, outbound quantity, transfer quantity and passenger flow quantity, show the dynamic changes in the passenger flow state every 15 min between 7:00 am and 9:00 am at morning peak hours on 15 January 2019. Meanwhile, comparative analysis is performed between the estimated values and actual data, as presented in Table 4.

**Figure 5:**
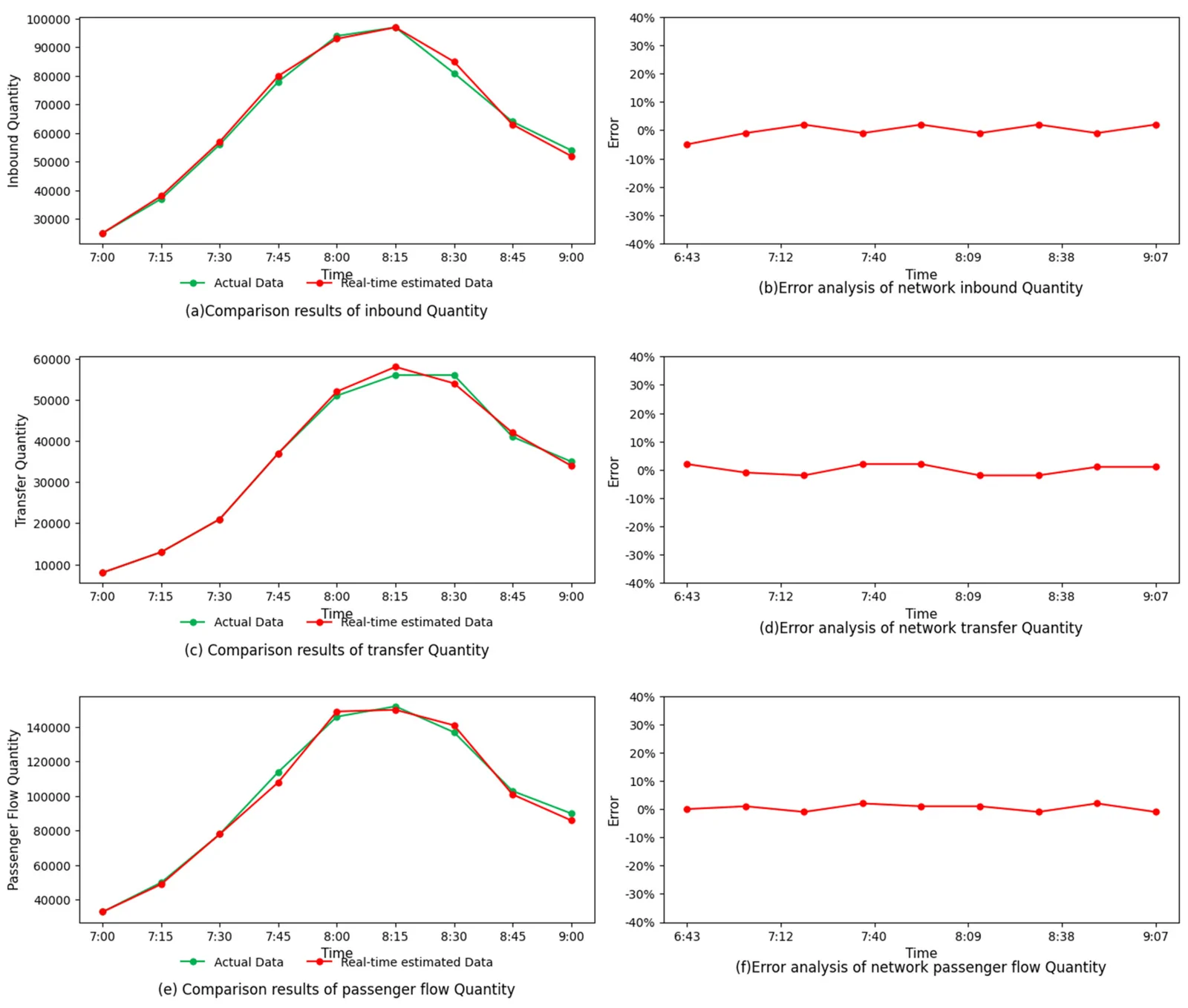
Comparison of estimation results of network-level passenger flow state and actual data.

As can be observed from Figure 5a,c,e, there is a certain error between the network-level inbound quantity, outbound quantity, transfer quantity, passenger flow quantity and corresponding actual data from 7:00 to 9:00 at morning peak hours, but the overall goodness of fit remains high. By comparing the errors of different types of indicators from 7:00 to 9:00 at morning peak hours, it can be found from Figure 5b,d,f that the errors of different network-level indicators falls within 3% for the majority of cases in daily URT operation periods.

**Figure 6:**
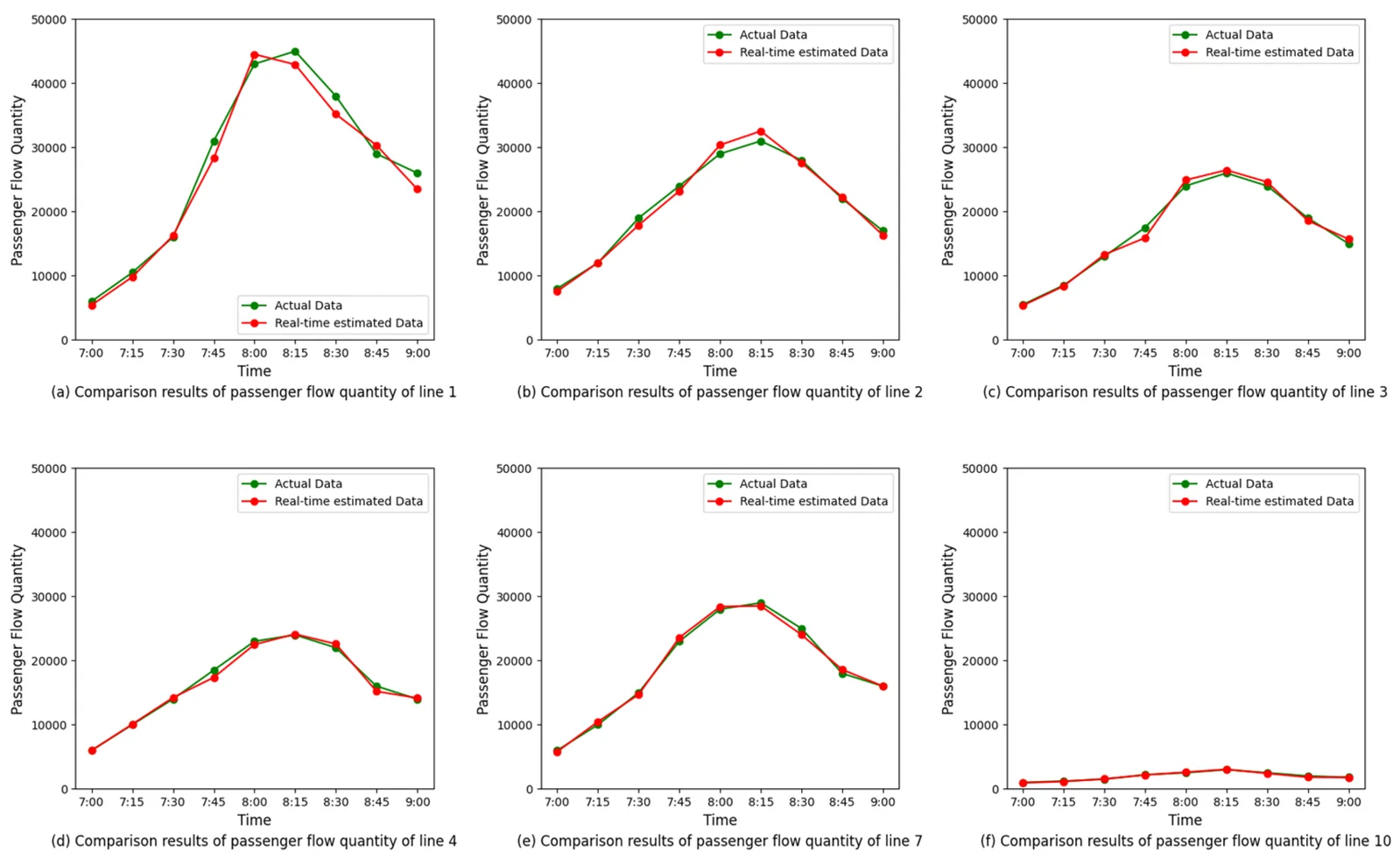
Comparison of estimation results of different lines and actual data.

**Figure 7:**
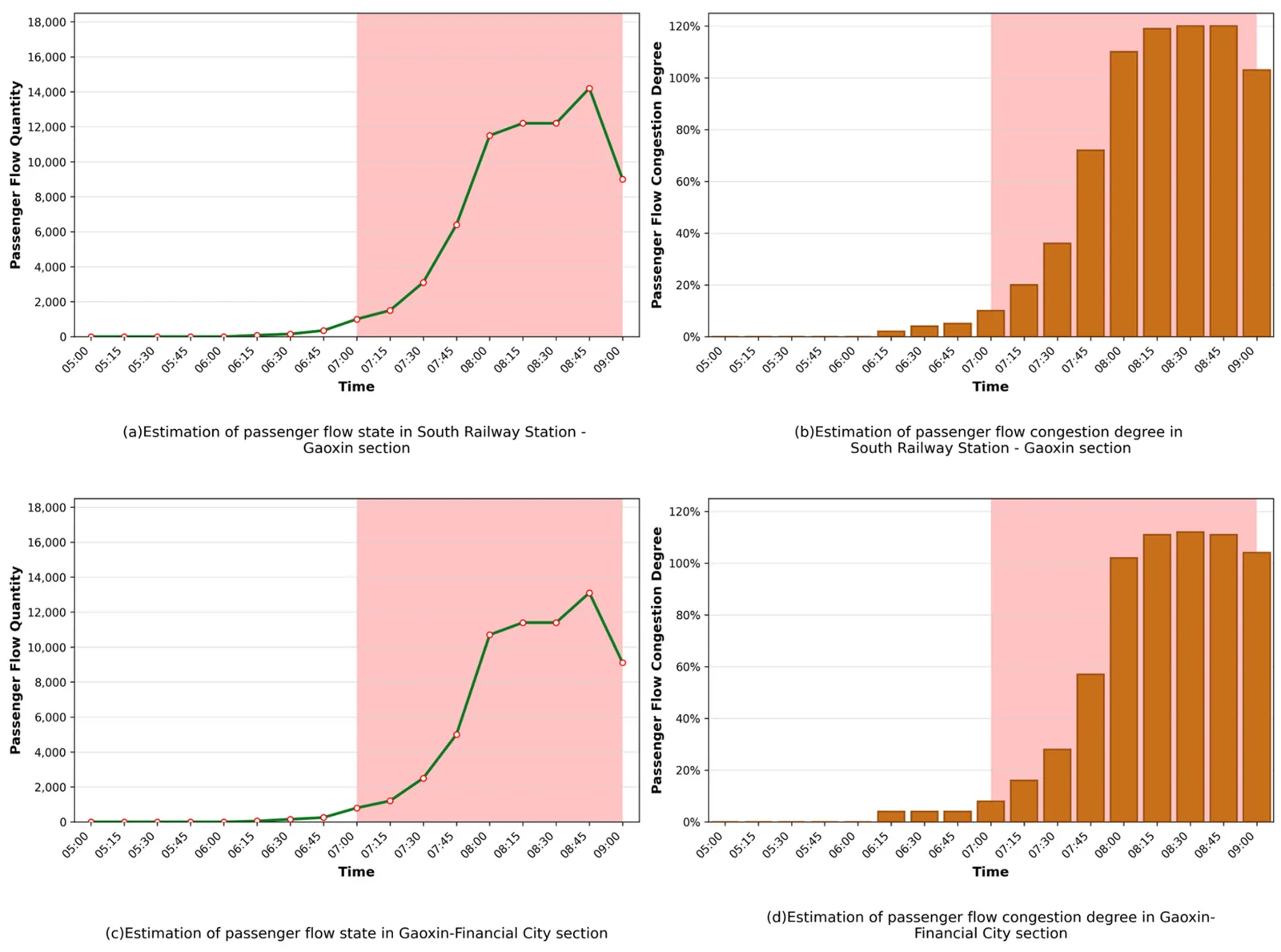
Changing trend of passenger flow quantity and congestion degree on the typical sections at morning peak hours.

**Figure 8:**
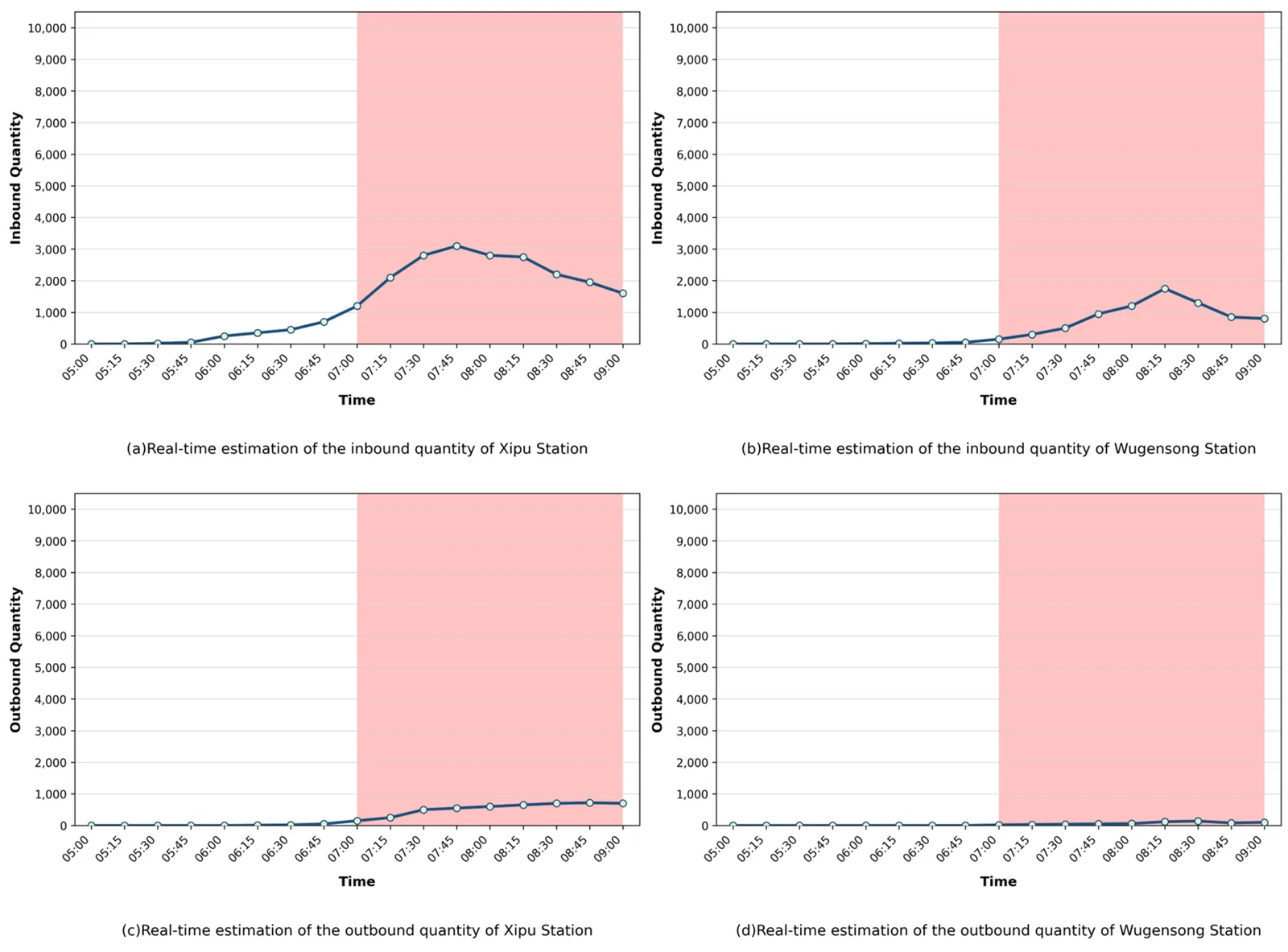
Changing trend of inbound passenger flow and outbound passenger flow at typical stations at morning peak hours.

Consistent patterns are identified in the passenger flow estimation results and corresponding analytical conclusions across all layers (network, line, section, and station layers). Accordingly, this section only presents the comparative results of the line-level estimated passenger flow indicators against the actual data, as shown in Figure 6. Herein, the indicator of the passenger flow congestion degree is introduced to intuitively visualize the temporal variations in passenger flow on typical URT sections, as plotted in Figure 7. The passenger flow congestion degree is a standardized quantitative sub-index of the passenger flow state, which measures the severity of passenger crowding in confined URT spaces (network, line, section, station, platform, transfer passages, train carriages). Moreover, the time-varying passenger flow trends at representative stations are provided in Figure 8.

By comparing the errors of different network-level indicators including network-level inbound quantity, outbound quantity, transfer quantity and passenger flow quantity, from 7:00 to 9:00, it can be found that the overall error of different network-level indicators falls within 3% for the majority of cases in daily URT operation periods, with a small proportion of outliers exceeding this threshold (due to the instantaneous crowding at transfer stations, unbalanced short-term passenger travel demand fluctuations, etc.), which further validates the better accuracy of the estimation results. Meanwhile, for all estimated indicators at the line level, namely the line-level inbound quantity, outbound quantity, transfer quantity and passenger flow quantity, their estimation errors compared with the actual data are approximately 3%. The estimated indicators of the section layer (including sectional passenger flow quantity and sectional congestion degree) are affected by the number of waiting passengers at stations and passengers’ varying comfort demands. The error between the estimated indicators of the section layer and the actual data stays below 10%. The estimated indicators of the station layer (including inbound quantity of stations, outbound quantity of stations, and transfer quantity of transfer stations) have strong correlations with the distance from auto gates to platforms, indoor facility arrangement, statistical time interval as well as the implementation of passenger flow control strategies. The error between the estimated indicators of the station layer and actual data also falls within 10%.

### 4.3. Evaluation Criteria and Benchmark Model

The dataset used in this study as the original passenger flow data includes several consecutive months of Chengdu Metro AFC data from 10 October 2018 to 20 January 2019, which encompasses six lines and 156 stations. In order to extract the inbound and outbound passenger flow, the inbound/outbound times are transformed into minutes, ranging from 0 to 1080, representing 05:00 to 23:00. Each metro station has a unique station ID, by which inbound/outbound passenger flow time series are extracted at a 15 min time granularity. The total data volume is 1.4 × 10^10^ with 72 time steps per day. In the experimental process, the dataset used in this study is divided into training, validation, and testing sets in a ratio of 7:2:1. Prior to training, all data is Min–Max normalized. This study adopts the Mean Absolute Error (*MAE*), Root Mean Square Error (*RMSE*), and Mean Absolute Percentage Error (*MAPE*) as the evaluation criteria for the proposed model. Their equations are as follows, where *N* represents the total number of passenger flows, $y_{i}$ represents the actual value, and ${\hat{y}}_{i}$ represents the predicted value. The lower value of the criterion indicates the better performance of the model.

(21) $$\mathrm{RMSE}=\sqrt{\frac{1}{N}\sum_{i=1}^{N}{\left(y_{i}-{\hat{y}}_{i}\right)}^{2}}\;\mathrm{MAE}=\frac{1}{N}\sum_{i=1}^{N}\left|y_{i}-{\hat{y}}_{i}\right|\;\mathrm{MAPE}=\frac{100\%}{N}\sum_{i=1}^{N}\left|\frac{y_{i}-{\hat{y}}_{i}}{y_{i}}\right|$$

To further demonstrate the effectiveness of the proposed model, it is compared against the following benchmark models. The benchmark models are divided into two categories, namely models based on single-source data and models based on multi-sourced data. Except for the parameters inherent to each benchmark model, all other hyperparameters are set identical to those of the proposed model. The benchmark models are as follows: ARIMA, CNN, LSTM, GSN, Transformer, Conv-GCN, and DEAseq2seq. The evaluation metrics for different models at the network level are shown in Table 5, and the proposed model outperforms any benchmark model within the 15 min time granularity. Compared to the second-best-performing model, namely the DEASeq2Seq model, the proposed model reduces the MAE by 8.1%, RMSE by 10.7%, and MAPE by 17.2% within the 15 min time granularity, respectively. The experimental results for the network show the effectiveness of the proposed model in the URT network.

**Figure 9:**
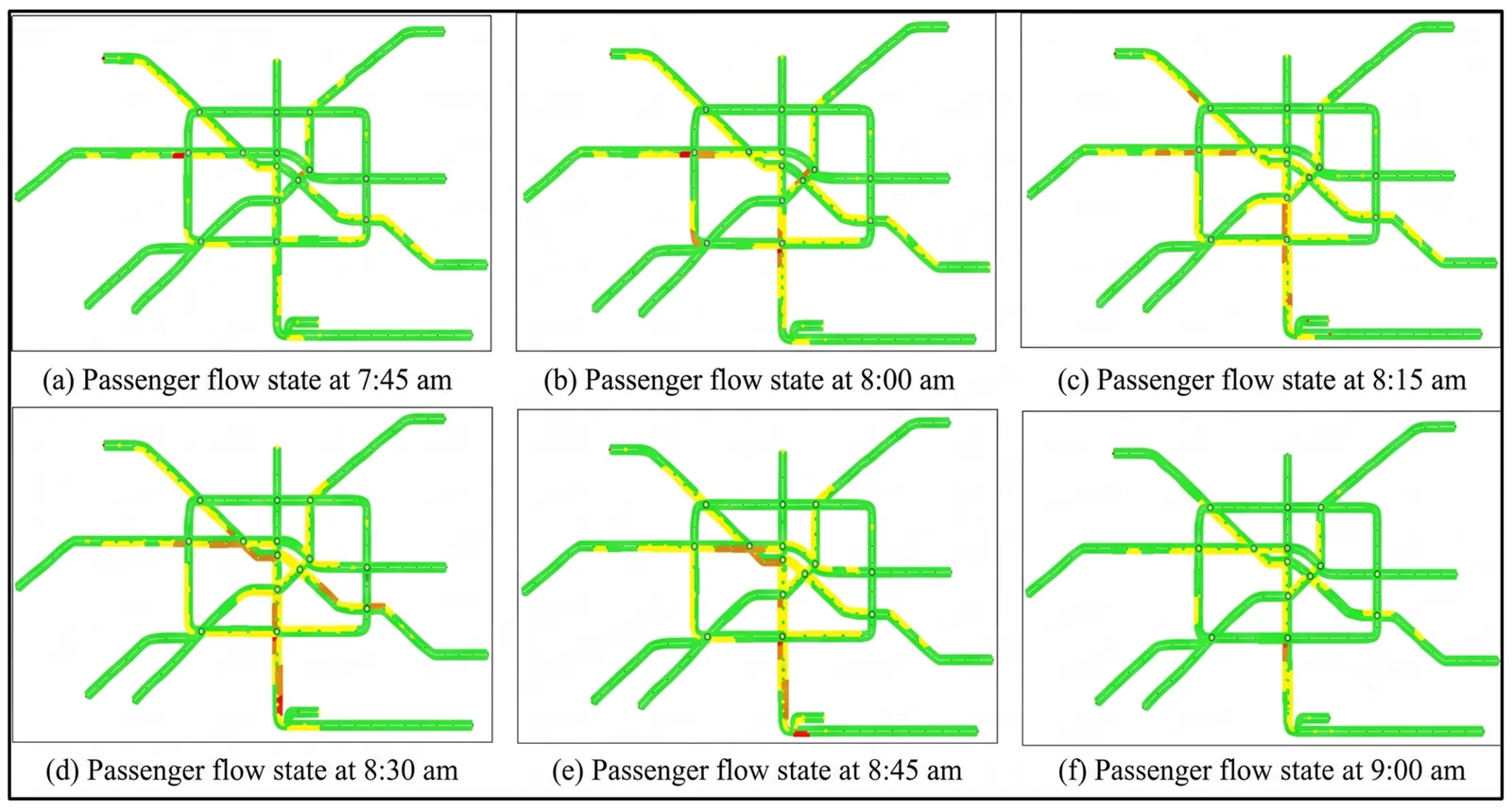
Estimated situation of network-level passenger flow state of network at morning peak hours in Chengdu Metro.

For the purpose of capturing real-time fluctuations in the passenger flow state of different lines across the whole network, it is essential to estimate the real-time changes in the passenger flow state in all lines (inbound quantity and outbound quantity of different stations, passenger flow quantity, congestion degree of different sections, etc.). In Figure 9, six time intervals at morning peak hours between 7:45 am and 9:00 am (7:45, 8:00, 8:15, 8:30, 8:45, 9:00) were chosen on 15 January 2019 and the corresponding estimated results of the passenger flow state at these time intervals are obtained in each line. The obtained estimated results support the Chengdu Metro in supervising the service quality and congestion level of trains in each line. Meanwhile, targeted congestion mitigation measures such as adjusting train departure frequency and supplementing running trains can be scientifically and accurately determined. In addition, real-time updates can be delivered as the passenger flow state shifts, which facilitates passengers to decide their travel time after learning the running conditions of urban rail transit.

## 5. Conclusions

In light of the uneven distribution of URT passenger flow, the transmission delay in the uploading process of AFC data and the practical demand for multi-source data application, this study investigates passenger flow estimation based on multi-source data. The main research results of this study are as follows:

(1) This study utilizes the multi-source data acquired from mainstream sensors such as through-beam photoelectric sensors and RFID/NFC sensors to accomplish the data fusion, estimation and calibration for the accuracy of the estimation results in the passenger flow estimation of the URT network. In the estimation process of the passenger flow state, the AD method is introduced and modified as the framework to achieve the fusion of multi-source data (including AFC data, mobile phone signaling data and historical passenger flow data) considering the heterogeneity of multi-source data and the complicatedness of multi-source heterogeneous data fusion.

(2) This study establishes a multi-source data-driven estimation model and improved error layered optimization algorithm. The real-world application of the model and algorithm proves its effective performance in terms of accuracy and stability. Furthermore, the experimental results for the network demonstrate the superiority of the proposed model. For the URT network, compared to the best benchmark model, the proposed model reduces the MAE by 8.1%, RMSE by 10.7%, and MAPE by 17.2% within the time granularity of 15 min respectively.

In future research, it is worthwhile considering fusing more data types derived from different kinds of sensors such as Wi-Fi data, surveillance video data, meteorological data, air quality data, timetable data and others to further enhance the accuracy of passenger flow estimation, especially in some special scenarios with irregularly and rapidly changing passenger flow, such as new line expansion, large events, emergencies, etc. In addition, multi-city URT network tests could be carried out in future research to prove the proposed model’s transferability. Moreover, different transit modes could be taken into consideration as well to achieve better transfer connectivity and service level of public transportation.

## Figures and Tables

**Table 1:** Summary of parameters and variables.

Notations	Definitions
**Sets**	
V	Subset of stations in the URT network
E	Subset of sections in the URT network
$W_{o}$	Set of origin–destination (OD) pairs starting from origin o, o∈V
W	Set of all OD pairs in the URT network
$R_{w}$	Set of all K shortest routes for OD pairs w=(o,d)
**Indexes**	
o	Travel origin station in the URT network, o∈V
d	Travel destination station in the URT network, d∈V
w	Member in the set W of OD pairs, w∈W
r	Member in the set *R* of routes, r∈R
a	Member in the set E of sections, a∈E
N	Number of samples
**Input data**	
$\bar{X_{o}^{t}}$	Sample data of inbound passenger flow at station o in time period *t* based on historical passenger flow data, o∈V
$\bar{P_{ow}^{t}}$	Selection ratio of OD pairs w∈W in all OD pairs departing from the station o based on historical passenger flow data, $0\leq \bar{P_{ow}^{t}}\leq 1$
$\bar{P_{wr}^{t}}$	Selection ratio of routes r∈R in OD pairs w∈W based on historical passenger flow data, $0\leq \bar{P_{wr}^{t}}\leq 1$
$\bar{X_{a}^{t}}$	Sample data of passengers passing through the section a∈E collected by mobile phone signaling data
$\bar{Z_{o}^{t}}$	Sample data of outbound passenger flow at station o in time period t based on historical passenger flow data, o∈V
$\delta_{ra}$	If the route r within the OD pairs passes the section a, then δ = 1; otherwise, δ = 0. r∈R, a∈E
$t_{o}^{in}$	Inbound travel time at station o in the URT network, o∈V
$t_{o}^{out}$	Outbound travel time at station o in the URT network, o∈V
$t_{o}^{tran}$	Transfer travel time at station o in the URT network, o∈V
$\pi_{ow}^{t}$	OD proportion of OD pair w departing from the station o in time period t before normalization, o∈V, w∈W
$t_{a}$	Travel time of section a in the URT network, a∈E
θ	Penalty coefficient θ of passenger travel route r passing the transfer station, θ > 1, r∈R
$\delta_{rtran}$	If passenger travel route *r* traversing the transfer station $\delta_{rtran}$ = 1; otherwise, $\delta_{rtran}$ = 0
**Variables**	
$X_{o}^{t}$	Estimated inbound quantity at o station in time period t in the URT network, o∈V
$P_{ow}^{t}$	Estimated selection ratio from station o to OD pair *w* in time period t in the URT network, o∈V, w∈W
$X_{w}^{t}$	Estimated OD distribution of OD pair w∈W in time period *t* in the URT network
$P_{wr}^{t}$	Estimated selection ratio of route r∈R in OD pair w∈W in time period t in the URT network
$X_{r}^{t}$	Estimated passenger flow of route r in time period *t* in the URT network, r∈R
$Z_{o}^{t}$	Estimated outbound quantity at o station in time period t in the URT network, o∈V
$X_{a}^{t}$	Estimated passenger flow of section a in time period *t* in the URT network, a∈E

**Table 2:** Comparative analysis of estimation results of network-level passenger flow state and actual data.

Date	Estimated Inbound Quantities/ Actual Data	Error	Estimated Outbound Quantities/ Actual Data	Error
10 October 2018	566,729/618,067	−8.31%	453,220/420,559	7.77%
11 October 2018	550,842/609,909	−9.68%	439,003/411,442	6.70%
12 October 2018	539,801/500,985	7.75%	430,866/413,631	4.17%
13 October 2018	270,203/299,819	−9.88%	201,755/185,614	8.70%
14 October 2018	208,112/224,960	−7.49%	154,662/136,102	13.64%
15 October 2018	590,375/543,508	8.62%	463,067/416,760	11.11%
16 October 2018	560,453/620,893	−9.73%	448,069/429,107	4.42%
17 October 2018	557,636/627,823	−11.18%	444,869/400,382	11.11%
18 October 2018	542,715/501,559	8.21%	421,853/451,382	−6.54%
19 October 2018	557,819/510,193	9.33%	441,390/494,356	−10.71%
20 October 2018	275,380/301,164	−8.56%	206,865/182,041	13.64%
-	-	-	-	-

**Table 3:** Ground-truth AFC data samples.

In Station Code	In Station Time	Outstation Code	Outstation Time	Card Number	Card Type
0137	08:06:06	0346	09:15:03	001641823	22
0121	07:55:16	0420	08:55:14	002119204	22
0310	07:52:10	0737	08:20:01	003177195	22
1026	07:45:38	0226	08:31:22	003652895	22
0225	08:03:44	0729	08:51:29	004804144	22
0738	08:25:11	1024	08:47:21	004996383	22
0431	08:10:16	0140	08:37:58	005597524	22
-	-	-	-	-	-

**Table 4:** Comparative analysis of estimation results of network-level passenger flow state and actual data.

**Period**	**Estimated Inbound** **Quantities/** **Actual Data**	**Error**	**Estimated Outbound** **Quantities/** **Actual Data**	**Error**
7:00	24,084/24,298	−0.88%	8542/8669	−1.46%
7:15	37,701/37,030	1.81%	14,353/14,170	1.29%
7:30	55,830/56,010	−0.32%	23,868/23,204	2.86%
7:45	79,045/77,789	1.61%	33,732/34,345	−1.79%
8:00	94,174/94,288	−0.12%	58,042/57,002	1.82%
8:15	98,072/96,541	1.59%	83,287/81,458	2.25%
8:30	80,859/81,234	−0.46%	100,974/102,916	−1.89%
8:45	64,106/62,849	2.00%	110,098/107,939	2.00%
9:00	53,916/54,820	−1.65%	70,870/70,461	0.58%
-	-	-	-	-
**Period**	**Estimated transfer quantities/** **Actual Data**	**Error**	**Estimated passenger flow quantities** **/Actual Data**	**Error**
7:00	7903/7763	1.80%	31,987/32,061	−0.23%
7:15	13,195/13,256	−0.46%	50,895/50,286	1.21%
7:30	20,671/21,091	−1.99%	76,501/77,101	−0.78%
7:45	37,207/36,549	1.80%	116,252/114,338	1.67%
8:00	51,985/50,968	2.00%	146,159/145,256	0.62%
8:15	55,332/56,318	−1.75%	153,404/152,859	0.36%
8:30	54,805/55,997	−2.13%	135,664/137,231	−1.14%
8:45	41,292/40,857	1.07%	105,398/103,706	1.63%
9:00	35,136/34,809	0.94%	89,052/89,629	−0.64%
-	-	-	-	-

**Table 5:** Evaluation metrics for different benchmark models at the network level.

Models	Data Input	MAE	RMSE	MAPE (%)
ARIMA	Only AFC Data	92.4	98.7	9.62
CNN	Only AFC Data	86.8	93.2	9.07
LSTM	Only AFC Data	81.9	88.1	8.51
GCN	Only AFC Data	87.9	94.3	8.94
Transformer	Only AFC Data	84.6	90.8	8.72
Conv-GCN	AFC Data + Historical Passenger Flow Data + Mobile Phone Signaling Data	28.2	32.6	1.89
DEASeq2Seq	AFC Data + Historical Passenger Flow Data + Mobile Phone Signaling Data	23.4	27.2	0.87
Proposed Model	AFC Data + Historical Passenger Flow Data + Mobile Phone Signaling Data	21.5	24.3	0.72

## Data Availability

The data presented in this study is available on request from the corresponding author due to copyright limitations of the third-party original datasets.
